# Curcumin-Mediated Photodynamic Treatment Enhances Storage Quality of Fresh Wolfberries via Antioxidant System Modulation

**DOI:** 10.3390/foods14162843

**Published:** 2025-08-16

**Authors:** Yan-Fei Shen, Wen-Ping Ma, Run-Hui Ma, Kiran Thakur, Zhi-Jing Ni, Wei Wang, Zhao-Jun Wei

**Affiliations:** 1 School of Biological Science and Engineering, Specialty Food Nutrition and Health Innovation Team of Ningxia Hui Autonomous Region, North Minzu University, Yinchuan 750021, China; 2 School of Food and Biological Engineering, Hefei University of Technology, Hefei 230601, China

**Keywords:** wolfberry, curcumin-mediated photodynamic treatment, postharvest preservation, antioxidant enzymes

## Abstract

Photodynamic inactivation (PDI) is an innovative non-thermal sterilization and preservation method that has recently emerged as a safe, effective, cost-effective and environmentally sustainable alternative for biomedical applications. Curcumin (Cur), a commonly used food additive, possesses photosensitizing properties. In this study, we investigated the effect of curcumin-mediated photodynamic treatment (Cur-PDT) on the preservation of fresh wolfberries. Our experimental data revealed that a Cur-PDT treatment using a cur concentration of 500 μmol/L for 30 min, with 20 W irradiation, achieved the best preservation effect on fresh wolfberries. This intervention significantly slowed the decline in post-harvest hardness and delayed the progression of decay. It also reduced the accumulation of malondialdehyde (MDA), hydrogen peroxide (H_2_O_2_) and superoxide anion (•O_2_^−^). Notably, at day 3, the enzymatic activities of catalase (CAT) and ascorbate peroxidase (APX) in Cur-PDT-treated wolfberries were 1.12 and 1.88 times higher, respectively, than those in the control group. These elevated enzyme activities promoted the biosynthesis and recycling of ascorbic acid (AsA) and glutathione (GSH), leading to their substantial accumulation under oxidative stress conditions. By modulating the antioxidant defense system, Cur-PDT has the potential to extend the shelf-life of post-harvest wolfberries and enhance their overall quality attributes, thereby maintaining physiological homeostasis during storage.

## 1. Introduction

*Lycium barbarum* L., commonly known as wolfberry, is a fleshy, oblong, red-orange fruit native to Asia and predominantly found in dry and semi-dry regions. Wolfberries are rich in nutrients and bioactive components, such as carotenoids, phenols, flavonoids, vitamins, minerals, trace elements, and wolfberry polysaccharides. These compounds proven to possess immune-boosting, anti-aging, anti-cancer, antioxidant, and other medicinal properties [1].

Wolfberries are highly susceptible to decay after harvesting due to thin skin, exposure to high temperatures, active metabolism, latent fungal infections, and mechanical damage, all of which lead to fruit rupture and weight loss, ultimately resulting in rapid deterioration. Currently, preservation methods for wolfberries include cryopreservation, chitosan coating, hydrogen sulfide fumigation, and salicylic acid immersion [2]. However, these traditional preservation techniques have notable limitations. Cryopreservation is energy-intensive and relies heavily on cold-chain transportation. Chitosan coating can alter the fruit surface permeability, potentially affecting flavor release. Hydrogen sulfide fumigation poses risks of gas residue and secondary contamination. Salicylic acid immersion requires a strict operating environment and may lead to nutrient loss. Given these challenges, the development of novel green preservation technologies that do not have chemical residues during post-harvest handling is imperative.

Photodynamic technology, characterized by its safety, cost-effectiveness, energy-efficiency, and ability to preserve nutritional and sensory attributes while avoiding harmful by-products, has become a significant method for food preservation [3]. Its main mechanism involves using triple-state photosensitizers and triplet oxygen molecules to generate singlet oxygen (^1^O_2_) [4]. This electrophilic ^1^O_2_ reacts with oxidation-sensitive biomolecules (such as unsaturated fatty acids, proteins, nucleic acids, etc.) [5], disrupting cellular membrane structure and functional homeostasis. This disruption compromises the cellular uptake of physiologically active molecules, ultimately leading to impaired cellular absorption and cell death [6,7]. Recently, this technology has demonstrated considerable applicability within the domain of food safety, notably through photodynamic inactivation, which has been demonstrated to effectively inhibit lipid oxidation in refrigerated oyster samples [8]. In riboflavin-mediated photodynamic technology, riboflavin photosensitization has been found to enhance post-harvest kiwifruit resistance to gray mold [9]. The antimicrobial efficacy of various photosensitizer-mediated photodynamic techniques has been investigated across food products, including orange juice [10], freshly cut pineapple [11], apple juice [12], and kiwifruit [9]. As a novel sterilization method, photodynamic technology offers unique advantages and promising development prospects compared to other sterilization methods.

Curcumin, as a natural polyphenolic photosensitizer, combines broad-spectrum antimicrobial and antioxidant activities. Its photosensitization reaction generates single-linear oxygen, which penetrates cell membranes and destroys the DNA structures of pathogenic bacteria. Additionally, curcumin delays fruit browning by regulating the phenolic metabolism pathway. Unlike synthetic photosensitizers, curcumin is biodegradable and aligns with food safety standards. Curcumin-mediated PDT has shown potential in preserving fresh-cut fruits [13], including fresh-cut pineapple [11], psyllium [14], fresh jujube [15], and strawberries [16]. As a photosensitizer, curcumin exhibits phototoxicity against bacteria, fungi, and yeast [17]. Studies have demonstrated its efficacy against *Penicillium* [18], *Staphylococcus aureus* [19], and *Listeria monocytogenes* [20]. Given its dual role as both an antimicrobial and antioxidant agent, the present study employs curcumin as a photosensitizer.

Although the application of cur-mediated PDT for post-harvest preservation and wolfberry preservation and quality improvement of wolfberries has not been previously explored previously, this study investigates the bactericidal, bacteriostatic, and preservative effects of this novel non-thermal photodynamic technology on pathogenic bacteria that attack fresh wolfberries. This study systematically analyzes the impact of this technology on the antioxidant properties of the fruit, thereby unraveling the underlying biological mechanisms.

## 2. Materials and Methods

### 2.1. Materials

Fresh wolfberry ‘608’ fruits were sourced from the Institute of Wolfberry Research, Ningxia Academy of Agricultural and Forestry Sciences (Yinchuan, Ningxia, China). The freshly harvested wolfberries were promptly transported to the laboratory within 2 h in sterile, sealed containers. The wolfberries were carefully selected based on uniform morphological characteristics, including size, color, shape, and stage of maturity. Stringent quality control measures were employed to exclude any samples with visible insect/disease infestation, as well as those with mechanical damage.

### 2.2. Photosensitizer and Illumination Source Instrument

Following the method of preparation of Song et al. [18], a 1000 μmol/L of cur solution was prepared, containing 1% ethanol and 2 mL Tween 80. All preparation steps were conducted under light-protected conditions, and the solution was stored at 4 °C for subsequent use. The illumination source instrument consisted of a 20 W, 420 nm violet lamp, an illumination box, and an exhaust fan.

### 2.3. Sample Treatment

Freshly harvested wolfberries were sterilized in a 1% (*v*/*v*) sodium hypochlorite solution for 30 s [21], then thoroughly rinsed with distilled water and air-dried at ambient temperature. The treated wolfberries were divided into four groups: the CK group (no treatment), the L group (light treatment only), the P group (sprayed with curcumin solution only), and the LP group (subjected to photodynamic therapy with 420 nm violet light at 20 W). Each group had three replicates, with 60 wolfberries in each replicate. We added this information in text. A total of 60 fresh wolfberry samples were arranged on an adjustable platform 20 cm away from the light source. A 500 μmol/L cur solution was uniformly applied to the surface of the samples, which were then irradiated for 30 min. All treated samples were stored in a light-proof PVC container at 28 °C and 38% humidity for 12 days. The spoilage rate, weight loss, and alterations in appearance of the fresh wolfberries were monitored every three days, starting from day 0. Untested fresh wolfberries were preserved at −80 °C for further analysis.

### 2.4. Evaluation of Fruit Decay Rate and Weight Loss Rate

For the assessment of decay, if the decayed area of the wolfberry was greater than or equal to 25% of the total area, it was considered decayed.

Throughout the storage period, fresh wolfberries were monitored at regular intervals, with the decay rate and weight loss rate evaluated every three days. The experiment was conducted in triplicate, with 60 fresh wolfberries per replicate. Results are presented as percentages.

Fruit rot rate was calculated as:

(1) $$\text{Fruit decay rate}=\frac{\text{number of decayed fruits}}{\text{total number of fruits}}\times 100\%$$

Fruit weight loss was quantified via gravimetric analysis, which involved periodic mass measurements of the samples under controlled environmental conditions:

(2) $$\text{Weight loss rate}=\frac{W_{1}-W_{2}}{W_{1}}\times 100\%$$

where W_1_ indicates the initial weight of fresh wolfberry after harvesting; and W_2_ indicates the weight of wolfberry measured every three days during storage.

### 2.5. Assessment of the Hardness of Fresh Wolfberry

In this experiment, the hardness of wolfberry fruits was assessed using a texture analyzer (TMS-PRO, New York, NY, USA), following the protocol of Elam et al. [22]. Hardness testing was conducted using a 2 mm diameter cylindrical probe (Brookfield TA-P-KIT2, Shanghai, China) applied to the equatorial region of wolfberries. Results were calculated as the average value from 10 wolfberries per test, with three replicates conducted to ensure reliability.

### 2.6. Evaluation of the Hue of Fresh Wolfberry

The instrumental color parameters (L*, a*, b*, c*, and H*) of the fruit peel were determined following the protocol established by Zhang et al. [2]. A total of 10 wolfberries were randomly selected, and spectrophotometric analysis was performed on the equatorial region of each fruit using a calibrated colorimeter (HP-2132, Shanghai, China) to obtain precise colorimetric parameters (L*, a*, b*, c*, H* values). The equations for c*, H*, and ΔE are given below:

(3) $$c^{*}\;=\;\sqrt{\text{∆}a^{*2}+{\text{∆}b}^{*2}}$$

(4) $$H^{*}=\arctan(\frac{b^{*}}{a^{*}})\;$$

(5) $$\text{△}E=\sqrt{{\left(L^{*}-L_{0}^{*}\right)}^{2}+{\left(a^{*}-a_{0}^{*}\right)}^{2}+{\left(b^{*}-b_{0}^{*}\right)}^{2}}$$

The settings L*, a*, and b* represent the color characteristics of wolfberry fruit on day N of storage, whereas $L_{0}^{*}$, $a_{0}^{*}$, and $b_{0}^{*}$ denote the initial color parameters of fresh wolfberry at the commencement of the storage period.

### 2.7. Analysis of Total Soluble Solids (TSS), Titratable Acid (TA), and Solid-Acid Ratio (TSS/TA) in Fresh Wolfberry

In this experiment, TSS and TA content in wolfberries were determined by modifying the method of Elam et al. [22]. Ten wolfberries were randomly selected, and their seeds were removed. The fruits were crushed into juice. The TSS and TA content were measured using a fruit glycosurfactometer (SAM-706AC, Seoul, Republic of Korea) according to the manufacturer’s instructions. The units of TSS, TA, and TSS/TA were expressed as percentages.

### 2.8. Quantitative Analysis of Soluble Protein, Flavonoid, and Betaine Contents in Fresh Wolfberry

To assess the total protein content in fresh wolfberry samples, we employed the BCA method, following the kit instructions provided by the manufacturer (Suzhou Comin Biotechnology Co., Ltd., Suzhou, China) [21]. A volume of 200 µL of supernatant was pipetted into a 96-well plate for measurement, and the absorbance was determined at 562 nm. Protein concentrations are expressed in mg/mL.

The flavonoids and betaine contents in fresh wolfberries were determined using commercial assay kits (Suzhou Comin Biotechnology Co., Ltd., Suzhou, China). For flavonoid determination [23], 0.1 g of wolfberry and 2 mL of 60% (*v*/*v*) anhydrous ethanol were added to a pre-cooled mortar and mixed in an ice bath. The mixture was then shaken at 60 °C for 2 h. After extraction, the supernatant was obtained by centrifugation at 25 °C and 10,000× *g* for 10 min. The flavonoids content was calculated by mixing the supernatant with the appropriate reagents, and the absorbance was measured at 510 nm after resting for 15 min at 25 °C.

For betaine determination, the sample was dried and passed through a 40-mesh sieve (Zhongbao Hardware Mesh Products Co., Ltd., Shijiazhuang, China). Then, 0.02 g of sample and 0.8 mL of water were added to the centrifuge tube and shaken at 60 °C for 30 min. Subsequently, 200 μL of the extract was transferred to a new centrifuge tube, mixed, and centrifuged at 25 °C and 10,000× *g* for 10 min. The supernatant was collected and allowed to react with the reagent. Finally, 200 μL of reaction mixture was added to a 96-well plate, and the absorbance was determined at 525 nm. Both flavonoid and betaine contents were expressed in mg/g.

### 2.9. Assessment of MDA and LOX Levels in Fresh Wolfberry

The MDA and LOX in the wolfberry were determined according to instructions given in the assay kit (Suzhou Comin Biotechnology Co., Ltd., Suzhou, China) [24]. The units of MDA and LOX were nmol/g and U/g, respectively.

### 2.10. Determination of H_2_O_2_ Content and Rate of •O_2_^−^ Production in Fresh Wolfberry

To evaluate the generation of reactive oxygen species (ROS) generation, the production rates of H_2_O_2_ and •O_2_^−^ production rates were quantified according to the protocol established by Yang et al. [25]. The H_2_O_2_ and •O_2_^−^ production rate kits were used according to the kit instructions. The concentration of H_2_O_2_ was expressed in μmol/g, while the production rate of •O_2_^−^ was expressed in nmol/g.

### 2.11. Evaluation of SOD, CAT, and POD Enzymatic Activities in Fresh Wolfberry

The activities of antioxidant enzymes in wolfberry were determined by referring to the method described by Liu et al. [26]. The enzymatic activities of SOD, CAT, and POD were measured according to the instructions provided in the assay kit. All the above enzyme activities were expressed in U/g.

### 2.12. Determination of ASA, GSH Contents and APX, GR, MDHAR, and DHAR Enzyme Activities in Fresh Wolfberry

The concentrations of ASA and GSH concentrations, as well as the enzymatic activities of APX, GR, MDHAR, and DHAR, were quantified using the protocols established by Lu et al. [27]. Following the specific kit instructions, AsA and GSH levels were quantified in μg/g, while the activities of APX, GR, MDHAR, and DHAR were expressed in units per gram of fresh weight (U/g).

### 2.13. Statistical Analysis

The results were expressed as the mean ± standard deviation and analyzed with one-way analysis of variance (ANOVA) in IBM SPSS Statistics (Version 27) (Armonk, NY, USA), followed by post-hoc t-tests for pairwise comparisons. Data visualization was performed using Origin 2022 (Northampton, MA, USA) and significance was indicated by * when *p <* 0.05.

## 3. Results

### 3.1. Appearance and Changes of Weight Loss, Hardness, and Decay of Fruit Wolfberry During Storage

**Figure 1:**
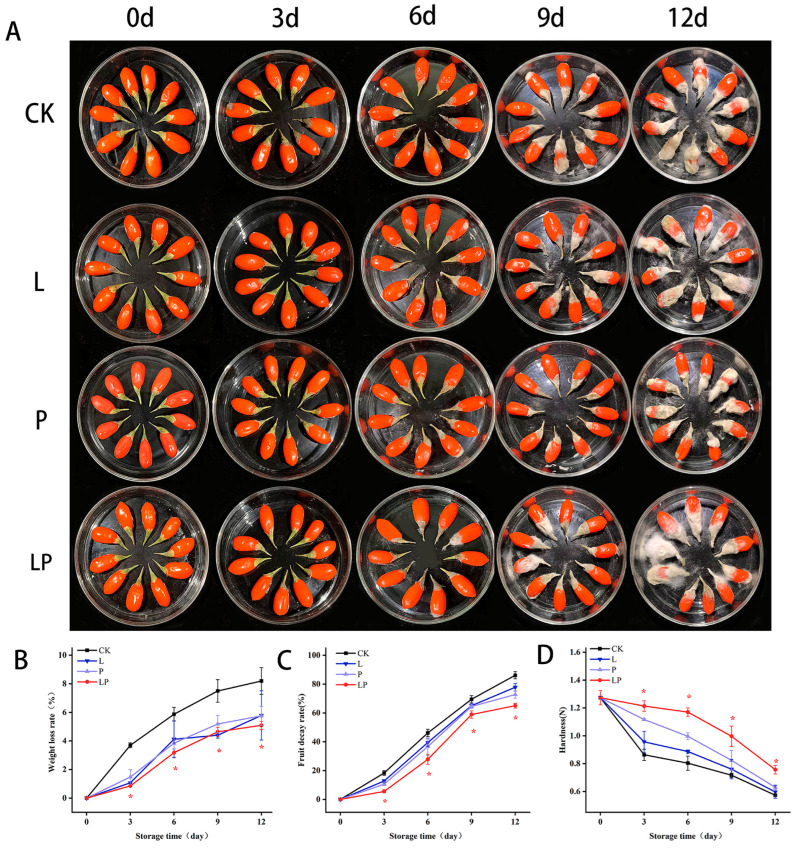
Effect of Cur-PDT on changes in appearance (**A**), weight loss (**B**), fruit decay rate (**C**), and hardness (**D**) of fresh wolfberry during storage period. CK, no treatment; L, light treatment only; P, sprayed with curcumin solution only; LP, subjected to photodynamic therapy with 420 nm violet light at 20 W. Data are presented as mean ± SD. Significant differences (*p* < 0.05) between the CK and LP groups are indicated by * at each sampling point.

During storage, weight loss and decay rates increased over time across all experimental groups, while fruit hardness decreased correspondingly. Figure 1A illustrates the impact of Cur-PDT treatment on the visual characteristics of fresh wolfberries throughout the post-harvest storage period. The rate of desiccation in fresh wolfberries followed an upward trajectory with prolonged storage (Figure 1B). The LP group consistently exhibited significantly lower weight loss than the CK group throughout the observation period. Specifically, the weight reduction in the LP group was 45.7% lower than that in the CK group on day 9 and 45.87% lower on day 12. Figure 1C indicates that the decay rate also increased over time during storage. The LP group exhibited 15.20% and 18.75% lower weight loss than the CK group on day 9 and day 12, respectively. Figure 1D reveals that the hardness of freshly harvested wolfberry fruit progressively declined over the storage period. However, LP group wolfberries maintained consistently higher hardness values than the CK group throughout the observation period.

### 3.2. Color Changes

**Figure 2:**
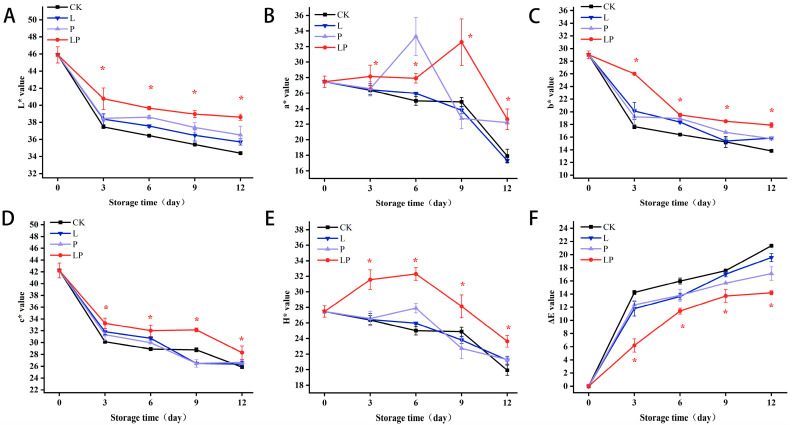
Effect of Cur-PDT on L* (**A**), a* (**B**), b* (**C**), c* (**D**), H* (**E**) and ∆E (**F**) of fresh fruit wolfberry during storage period. CK, no treatment; L, light treatment only; P; sprayed with curcumin solution only; LP, subjected to photodynamic therapy with 420 nm violet light at 20 W. Data are presented as mean ± SD. Significant differences (*p* < 0.05) between the CK and LP groups are indicated by * at each sampling point.

During the storage of wolfberries, the color parameters L*, a*, b*, c*, and H* exhibited declining trends, while the overall color difference ΔE revealed an increasing trend. As depicted in Figure 2A, the L* value in the LP group increased by 8.81% on day 3 and by 12.21% on day 12 compared to the CK group. Figure 2B further illustrates that a* values generally declined across all experimental groups, with the LP group consistently displaying significantly lower a* values than the CK group throughout the observation period. The b* value also declined during storage, with the LP group demonstrating a 31.92% increase in b* value relative to the CK group on day 3 (Figure 2C). In Figure 2D, the c* value increased by 10.16% in the LP group compared to the CK group on day 9. Figure 2E reveals a biphasic trend in H*, characterized by an increase followed by progressive attenuation throughout the storage period. The LP group achieved peak H* values on day 6 of storage, exhibiting a 1.29-fold elevation relative to the CK group. This pattern suggests a distinct temporal response mechanism between the experimental and control groups under storage conditions. Additionally, the LP group exhibited significantly lower ΔE values relative to the CK group throughout the experimental duration (Figure 2F).

### 3.3. Effect on TSS, TA, and TSS/TA

**Figure 3:**
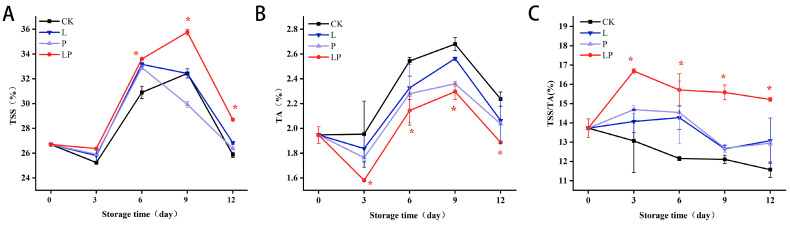
Effect of Cur-PDT on TSS (**A**), TA (**B**), and TSS/TA (**C**) of fresh fruit wolfberry during storage period. CK, no treatment; L, light treatment only; P; sprayed with curcumin solution only; LP, subjected to photodynamic therapy with 420 nm violet light at 20 W. Data are presented as mean ± SD. Significant differences (*p* < 0.05) between the CK and LP groups are indicated by * at each sampling point.

Figure 3A illustrates the decline in TSS content of fresh wolfberry with increasing storage time. The TSS concentrations in the LP group remained elevated relative to the CK group throughout storage, demonstrating statistically significant divergence (*p* < 0.05) from day 3 onward. Figure 3B indicates that the TA of postharvest wolfberry fresh fruits gradually decline during storage. While the TSS in both the CK and LP groups began to reduce from day 2, the TA content in Cur-PDT-treated fruits displayed a significantly slower degradation rate compared to the other groups. Figure 3C reveals the progressive increase in the sugar-acid ratio in postharvest fresh wolfberries during storage. Notably, Cur-PDT-treated wolfberry samples maintained a higher sugar–acid ratio than the CK group throughout the experimental period, with statistically significant differences (*p* < 0.05).

### 3.4. Effects on Flavonoids and Betaine Content

**Figure 4:**
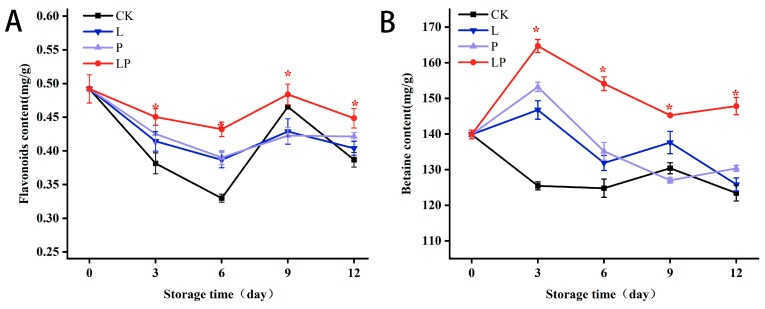
Effect of Cur-PDT on flavonoids (**A**) and betaine (**B**) of fresh fruit wolfberry during the storage period. CK, no treatment; L, light treatment only; P, sprayed with curcumin solution only; LP, subjected to photodynamic therapy with 420 nm violet light at 20 W. Data are presented as mean ± SD. Significant differences (*p* < 0.05) between the CK and LP groups are indicated by * at each sampling point.

Figure 4A demonstrates the dynamic variations in flavonoid content during storage. The LP group maintained elevated concentrations relative to the CK group. Specifically, the LP group exhibited 31.02% higher flavonoid content than the CK group on day 6, progressing to a 1.15-fold elevation by day 12 of storage. Figure 4B reveals a sustained elevation in betaine concentration in the LP group compared to the CK group throughout the storage duration. Quantitative analysis showed that the LP group had 23.50% and 19.78% higher betaine levels than the CK group on storage days 6 and 12, respectively.

### 3.5. Effects on Soluble Protein

**Figure 5:**
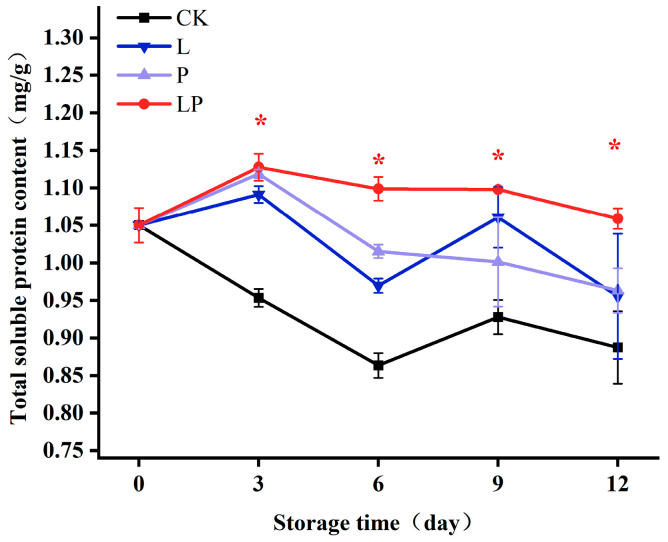
Effect of Cur-PDT on soluble proteins of fresh fruit wolfberry during storage period. CK, no treatment; L, light treatment only; P; sprayed with curcumin solution only; LP, subjected to photodynamic therapy with 420 nm violet light at 20 W. Data are presented as mean ± SD. Significant differences (*p* < 0.05) between the CK and LP groups are indicated by * at each sampling point.

As shown in Figure 5, the levels of soluble protein decreased steadily during storage. Throughout the entire storage period, the LP group consistently maintained markedly higher soluble protein levels compared with the CK group. Statistically significant differences between the two groups emerged starting from day 3. On day 3, the LP group had a 1.18-fold higher protein content than the CK group. By day 12, this ratio had increased to 1.32-fold, indicating an accelerated preservation effect of the LP treatment over time.

### 3.6. Effect on MDA, LOX, H_2_O_2_ and •O_2_^−^

**Figure 6:**
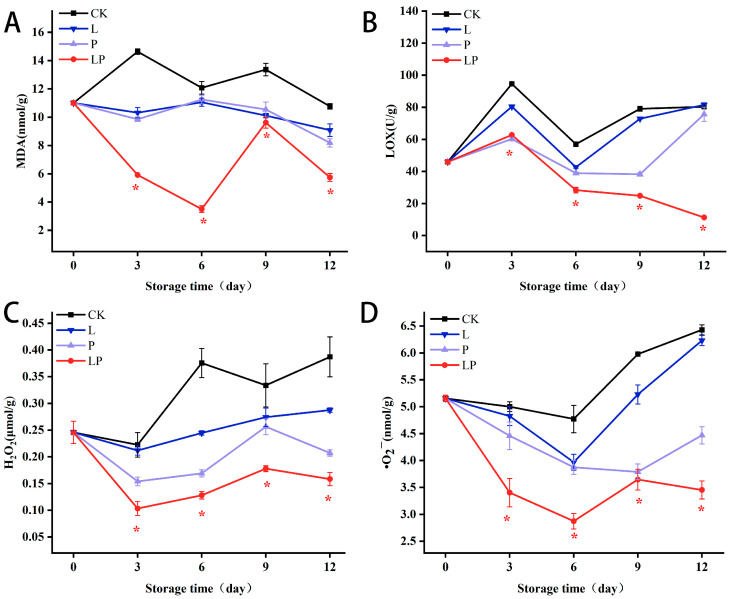
Effect of Cur-PDT on MDA (**A**), LOX (**B**), H_2_O_2_ (**C**), and •O_2_^−^ (**D**) activities of fresh wolfberry during storage period. CK, no treatment; L, light treatment only; P, sprayed with curcumin solution only; LP, subjected to photodynamic therapy with 420 nm violet light at 20 W. Data are presented as mean ± SD. Significant differences (*p* < 0.05) between the CK and LP groups are indicated by * at each sampling point.

Figure 6A demonstrates a statistically significant divergence in MDA concentrations between the LP and CK groups throughout the entire storage period. Notably, by day 12, the LP group exhibited a 28% reduction in MDA levels compared to the CK group. Figure 6B demonstrates a biphasic pattern in LOX activity, with the CK group consistently showing higher activity levels than the LP group throughout storage. Quantitative analysis indicated a 1.21-fold elevation in LOX activity within the CK group relative to the LP group at day 6. Figure 6C illustrated a consistent increase in H_2_O_2_ content over the entire storage duration, with the CK group showing a 1.2-fold increase over the LP group on day 3. Figure 6D demonstrated a progressive accumulation of •O_2_^−^ content during storage, with the LP group exhibiting a 46% reduction compared to the CK group by day 12.

### 3.7. Effects on the Activities of SOD, CAT, and POD

**Figure 7:**
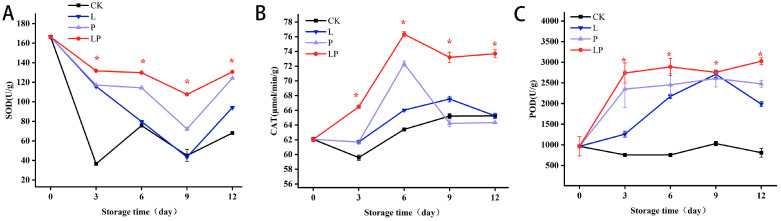
Effect of Cur-PDT on the activities of SOD (**A**), CAT (**B**), and POD (**C**) of fresh wolfberry during storage period. CK, no treatment; L, light treatment only; P, sprayed with curcumin solution only; LP, subjected to photodynamic therapy with 420 nm violet light at 20 W. Data are presented as mean ± SD. Significant differences (*p* < 0.05) between the CK and LP groups are indicated by * at each sampling point.

Figure 7A demonstrates a progressive increase in SOD activity throughout the storage period, with significant differences (*p* < 0.05) emerging from day 3 onwards. CAT activity also increased progressively during storage, with the LP group consistently exhibiting higher activity levels compared to the CK group (Figure 7B). After day 3, CAT activity in the LP group rose rapidly, peaking on day 6, while the CK group showed a relatively slower upward trend. Quantitative analysis indicated a 1.2-fold elevation in CAT activity in the LP group relative to the CK group on day 6. In Figure 7C, POD activity in fresh wolfberry fruit samples from both groups increased over time. The LP group reached its maximum POD activity at 12 days post-treatment, whereas the CK group achieved peak activity on day 9 before declining. By day 12 of storage, POD activity in the LP group was 37.35% higher than that in the CK group.

### 3.8. Effects on AsA and GSH Content, and Related Enzyme Activities in the AsA-GSH Cycle

**Figure 8:**
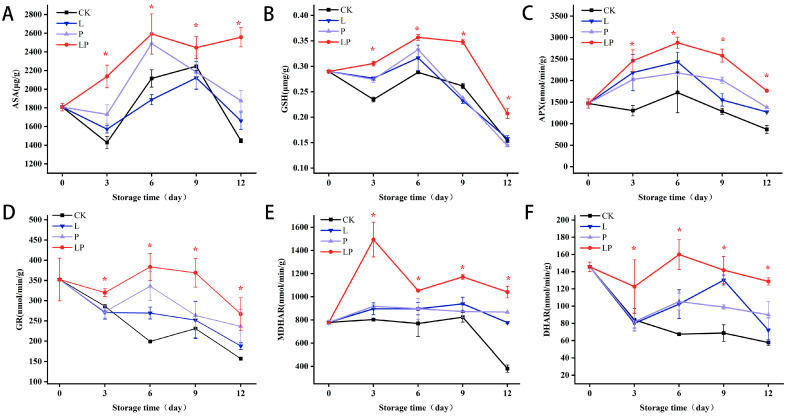
Effects of Cur-PDT on AsA (**A**) and GSH (**B**) contents and APX (**C**), GR (**D**), MDHAR (**E**), and DHAR (**F**) activities of fresh wolfberry during storage. CK, no treatment; L, light treatment only; P, sprayed with curcumin solution only; LP, subjected to photodynamic therapy with 420 nm violet light at 20 W. Data are presented as mean ± SD. Significant differences (*p* < 0.05) between the CK and LP groups are indicated by * at each sampling point.

Figure 8A showed that AsA content exhibited a biphasic fluctuation during storage, featuring an original rise followed by a decline. The maximum divergence in AsA levels between the LP and CK groups was observed on day 12, with the LP group showing a 1.5-fold increase relative to the CK group. GSH levels also followed a biphasic trend (Figure 8B), showing peak values at the midpoint of storage. Notably, on day 12, the CK group experienced a 28.28% reduction, whereas the LP group exhibited a more pronounced 46.21% decline in GSH content compared to their respective baseline values. As shown in Figure 8C, APX activity exhibited a storage-day-dependent pattern, with peak values on day 6 across all groups. The LP group showed a 35.47% increase compared to the CK group at this timepoint, maintaining consistently higher APX activity throughout storage. By day 12 of storage, APX activity in the LP group demonstrated a 1.90-fold elevation relative to the CK group. Figure 8D demonstrated a biphasic profile in GR activity within the LP group, with a peak at day 6 followed by progressive attenuation. The LP group maintained 36.69% higher GR activity than the CK group, which decreased by 26.16% over storage. MDHAR activity remained consistently elevated in the LP group (Figure 8E), with a marked 86% increase observed on day 3 compared to CK group. DHAR activity in the LP group demonstrated sustained elevation throughout storage (Figure 8F), peaking at day 6 and maintaining higher activity relative to CK thereafter.

### 3.9. Analysis of Correlation Between Quality Indexes and Antioxidant System of Fresh Wolfberry During Storage Period

**Figure 9:**
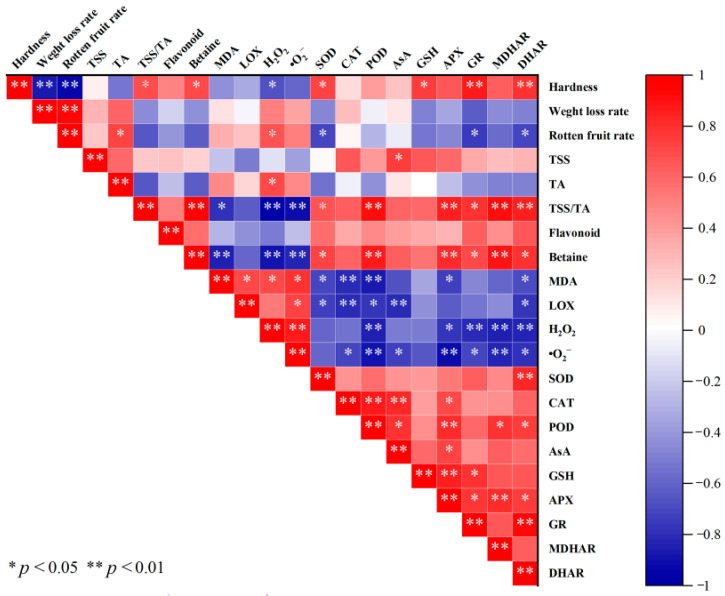
Correlation analysis of Cur-PDT on quality indexes and antioxidant system of fresh fruit wolfberry during storage period. **Note:** Data are presented as mean ± SD. Significant differences between the CK (no treatment) and LP groups (subjected to photodynamic therapy with 420 nm violet light at 20 W) are indicated at each sampling point.

The results presented in Figure 9 revealed that fruit hardness was significantly positively correlated with betaine content, GSH levels, SOD activity, and DHAR enzymatic function. Conversely, this parameter demonstrated a marked negative correlation with fruit rot incidence, weight loss progression, and H_2_O_2_ accumulation during postharvest storage.

## 4. Discussion

This study employed curcumin-mediated photodynamic technology to enhance the preservation of fresh wolfberries. The treatment significantly impacted multiple physiological parameters, including improving fruit quality, slowing senescence and deterioration processes, and enhancing stress tolerance.

Cur-PDT treatment significantly retarded wolfberry decay progression during storage (*p* < 0.05), concurring with findings reported by Seididamyeh et al. [16] on strawberry weight. The mechanism involves photodynamic therapy, which effectively reduces the number of spoilage-related pathogenic microorganisms through light-activated antimicrobial mechanisms. This effect may be attributed to the accumulation of phenolic compounds due to light-induced oxidative stress, which affects microbial degradation associated with fruit spoilage. This synergizes with the inherent antifungal properties of curcumin to jointly delay the spoilage process. Post-harvest water loss is a key factor affecting produce freshness and can lead to fruit shrinkage and spoilage [28]. The weight loss rate of wolfberry increased continuously during storage, while Cur-PDT treatment effectively reduced water loss, likely through cellular metabolism regulation and delayed fruit ripening [28]. Fruit hardness, an important indicator of ripening and preservation quality, decreased during storage. As shown in Figure 9, changes in hardness showed a significant negative correlation (*p* < 0.01) with the rates of decay and weight loss. The maintenance of hardness in the Cur-PDT treatment group could be attributed to reduced water evaporation, minimized cell collapse, inhibited pectinase activity, delayed cell wall degradation, or reduced microbial infestation, thereby preventing tissue disintegration [29].

Cur-PDT-treated wolfberry slowed the decline of L*, a*, b*, c* and H* values and the increase in ΔE during the storage, better preserving the fruit’s natural color characteristics. Dehydration of the fruit matrix and cuticle directly contributed to the decrease in L* values, which was mitigated by photodynamic treatments through the enhancement of humectancy mechanisms [30]. Studies have shown that increasing antioxidant enzyme activity may protect against postharvest fruit senescence and maintain high L* values [31]. Application of lecithin and salicylic acid effectively prolonged the postharvest storage of fresh wolfberries and sustained the a* value of the berries during storage. Notably, the synergistic effect of heat treatment and chitosan coating significantly increased the L* value of pickled wolfberry, improving their visual quality parameters [32]. This study confirms the synergistic effect of curcumin in improving the visual appeal of fresh-cut potato products. Its antioxidant and photo stabilizing properties improve the brightness and chroma of sliced tuber tissues, optimizing surface coloration [33]. Despite the photoinstability of curcumin solutions [34], high-concentration (1400 μM) treatment of jujubes did not alter color development. The maintenance of wolfberry coloration by Cur-PDT likely stems from its photostabilizing properties and antioxidant activity, which blocks browning chain reactions and binds biomolecules to optimize surface coloration [33]. This dual-pronged mechanism synergistically preserves fruit quality by inhibiting both spoilage microorganisms and oxidative damage.

TSS and TA are critical biochemical indicators for assessing the ripening stage and postharvest senescence of fruits [33]. TSS levels initially rose and then declined during storage, potentially linked to the fruit cell respiratory metabolism consuming endogenous sugars. Concurrent starch hydrolysis and conversion into sucrose, glucose, and other free sugars contribute to the observed elevation in TSS concentrations [34]. Subsequently, soluble sugars are utilized and metabolized as respiratory substrates during fruit senescence. Cur-PDT effectively inhibited the decline of TSS in postharvest wolfberry, postponed cellular degradation and starch breakdown, and delayed fruit ripening and aging. TA content was quantified via spectrophotometric determination of organic acid concentrations [33]. The accumulation of respiratory metabolites increases intracellular weak organic acids concentrations in fruit tissues, whereas pathogen-induced fungal colonization facilitates the efflux of these acids from the cell wall matrix. These combined biochemical processes contribute to the progressive elevation of total TA in postharvest fruits. Cur-PDT intervention attenuated the elevation of TA in wolfberries during prolonged storage, paralleling the effects of salicylic-acid-mediated postharvest treatment in the same fruit matrix [2]. The TSS/TA ratio serves as a key indicator of fruit quality, simultaneously assessing organoleptic properties and physiological maturity to evaluate sensory characteristics and developmental progression [35]. Cur-PDT treatment significantly retarded the decline in TSS/TA ratio throughout storage, aligning with effects observed in carvacrol-treated wolfberries [30]. Overall, Cur-PDT treatment largely maintained the quality characteristics of wolfberries.

Plants respond to biotic and abiotic stressors by synthesizing diverse phenolic compounds and flavonoids. Wolfberries are rich in proteins, vitamins, minerals, flavonoids, betaines, and other active components. Cur-PDT treatment significantly reduced the degradation of total proteins, betaines, and retained nutrients and bioactive compounds in the berries, thereby enhancing wolfberries’ defense capabilities.

ROS, including •O_2_^−^, H_2_O_2_, and lipid peroxidation end products like MDA, play crucial roles in cell signaling and metabolic pathways. However, excessive ROS can induce oxidative stress and damage cellular components [36]. Elevated H_2_O_2_, •O_2_^−^, and MDA concentrations are established biomarkers of ROS-mediated oxidative stress. During the 0–6 day observation period, the reduction in H_2_O_2_ and •O_2_^−^ levels in wolfberry controls may be attributed to the scavenging of some of the accumulated ROS by endogenous enzymatic antioxidant mechanisms inherent in wolfberry tissues. This defense system probably operates through redox reactions involving SOD and CAT, which collectively detoxify ROS and maintain redox balance during this critical developmental window. However, after day 6, reduced antioxidant enzyme activities and accelerated microbial proliferation led to increased ROS levels, inducing LOX and MDA increases, causing gradual oxidative damage to cellular constituents. Cur-PDT treatment, on the one hand, increased SOD and CAT enzyme activities to accelerate ROS scavenging; on the other hand, it reduced LOX [37] activity and MDA content, maintaining membrane lipid metabolism balance and delaying cell disintegration. Chen et al. [38] demonstrated that acidic electrolytic water treatment could reduce the accumulation of ROS by increasing the activity of antioxidant enzymes. Cur-PDT can inhibit cell membrane lipid peroxidation and alleviate ROS-induced oxidative stress through a dual mechanism: enhancing endogenous enzyme antioxidant pathways and attenuating microbial-mediated disruption of membrane integrity.

Plants primarily combat oxidative stress through an intrinsic defense system that includes antioxidant enzymes and small non-enzymatic antioxidants. Given this, we further investigated whether Cur-PDT reduces postharvest ROS accumulation in wolfberries by modulating the antioxidant defense system.

SOD, CAT, and POD constitute a core antioxidant enzyme system that regulates ROS homeostasis through a cascade reaction. SOD mediates the dismutation of •O_2_^−^ into H_2_O_2_ and O_2_. CAT subsequently decomposes H_2_O_2_ into H_2_O, while POD facilitates H_2_O_2_ scavenging through oxidation of phenolic/amine substrates and participates in electron transfer within the ascorbate-glutathione (AsA-GSH) cycle. Cur-PDT treatment increased SOD activity in wolfberry, while both CAT and APX activities were 1.2 and 1.9 times higher than those in the CK group, respectively. This regulation significantly reduced •O_2_^−^ and H_2_O_2_ accumulation. This may be due to the synergistic maintenance of H_2_O_2_ metabolic homeostasis by SOD and CAT, or the maintenance of membrane integrity by POD through lipid peroxidation inhibition. The supplementary results of Du et al. [37] showed that carbon-dot-mediated photodynamic treatments synergistically activated the functions of SOD, CAT, and POD, reducing intracellular superoxide anion and H_2_O_2_ levels. This maintained redox homeostasis and metabolic integrity, thus improving fruit quality.

The AsA-GSH cycle constitutes a core redox regulatory network through a four-step enzymatic reaction: reduction of H_2_O_2_ by APX using AsA to generate MDHA; regeneration of AsA by MDHAR using Nicotinamide Adenine Dinucleotide Phosphate Hydrogen (NADPH) as an electron donor; reduction of dehydroascorbic acid (DHA) to AsA by DHAR using GSH as a substrate; and regeneration of oxidized glutathione (GSSG) by GR-dependent NADPH reduction.

This cycle scavenges H_2_O_2_ through AsA-GSH redox and maintains cellular redox homeostasis [39,40]. Cur-PDT treatment significantly slowed ASA degradation in wolfberries, with the storage-end ASA retention rate reaching up to 2.3-fold of that of the control group (*p* < 0.05) [41]. This may be attributed to the blockage of H_2_O_2_ accumulation by elevated APX activity [42]; and the synergistic effect of MDHAR/DHAR to increase the regeneration efficiency of AsA. GSH dynamics showed that the GSH content of the LP group reached a peak on day 9, and then decreased, a phenomenon that was directly related to the enhancement of GR-mediated NADPH cycling and the inhibition of lipid peroxidation. This modulation effectively inhibited ASA oxidase activity by lowering the redox potential, increasing ASA-GSH cycling flux, and delaying oxidative stress aging.

A Pearson correlation analysis showed that wolfberry hardness was significantly negatively correlated with weight loss rate, decay rate, and hydrogen peroxide, and positively correlated with betaine, SOD, GR, and DHAR. Cur-PDT treatment reduced weight loss rate and membrane lipid peroxidation, thereby maintaining fruit hardness. Betaine, as an osmoregulatory core substance, was positively correlated with SOD, POD, APX, GR, MDHAR, and DHAR, and negatively correlated with MDA, H_2_O_2_, and superoxide anion. This indicates that the Cur-PDT treatment slowed down the degradation of nutritional quality by modulating the antioxidant enzyme system.

## 5. Conclusions

Cur-PDT effectively delayed wolfberry decay and mitigated post-harvest quality loss during storage. This treatment enabled wolfberries to retain higher color values (L*, a*, b*, and c*), as well as better hardness, TSS, and TSS/TA ratios, while also resulting in lower weight loss and TA values. Additionally, Cur-PDT slowed down the degradation of flavonoids, betaines, AsA, and GSH; while reducing the accumulation of MDA, LOX, H_2_O_2_, and •O_2_^−^. Furthermore, Cur-PDT enhanced the CAT, POD, MDHAR, and DHAR activities, thereby reducing oxidative damage in the fruits. By modulating enzyme activities and promoting antioxidant accumulation, Cur-PDT significantly boosts the antioxidant capacity of fresh wolfberries, effectively prolonging their freshness. This innovative approach not only offers a novel solution for wolfberry preservation, but also holds immense potential for widespread application in the wolfberry industry.

## Data Availability

The original contributions presented in this study are included in the article. Further inquiries can be directed to the corresponding authors.

## References

[1] Yu J., Yan Y.M., Zhang L.T., Mi J., Yu L.M., Zhang F.F., Lu L., Luo Q., Li X.Y., Zhou X. (2023). A comprehensive review of goji berry processing and utilization. Food Sci. Nutr..

[2] Zhang H.Y., Ma Z.M., Wang J.J., Wang P., Lu D.Y., Deng S.F., Lei H.L., Gao Y.F., Tao Y.Y. (2021). Treatment with exogenous salicylic acid maintains quality, increases bioactive compounds, and enhances the antioxidant capacity of fresh goji (*Lycium barbarum* L.) fruit during storage. LWT-Food Sci. Technol..

[3] Zhou Z.L., Li P.Z., Chen R.X., Cai X.Y., Zhang W.J., Fan P.H., Su J.Y. (2025). A Review of Curcumin-Mediated photodynamic bactericidal technology for food preservation: Limitations and improvement strategies. Food Microbiol..

[4] Li H.R., Ni Y.S., Zhao J.S., Li Y.M., Xu B.C. (2024). Photodynamic inactivation of edible photosensitizers for fresh food preservation: Comprehensive mechanism of action and enhancement strategies. Compr. Rev. Food Sci. Food Saf..

[5] Yang M., Chao H.J., Hou Z.H., Wang L.L., Xu W.Z., Zhao X. (2025). Antimicrobial activity of octyl gallate nanoemulsion combined with photodynamic technology and its effect on food preservation. Int. J. Food Microbiol..

[6] Dong L., Qin J.R., Tai L.Y., Mou K.Y., Liao X.J., Chen F., Hu X.S. (2022). Inactivation of *Bacillus subtilis* by curcumin-mediated photodynamic technology through inducing oxidative stress response. Microorganisms.

[7] Dong S.L., Chen L., Li S.J., Feng K.L., Liu G., Dong H., Xu G.Z., Ou H.J., Liu Y., Zhao Y. (2025). Antifungal activity of curcumin-mediated photodynamic inactivation against *Fusarium graminearum* on maize. Grain Oil Sci. Technol..

[8] Zhi J.J., Tang Q.J., Wu S.J., Kong B., Jiang J.L., Li Z.J., Wang Y.M., Xue C.H. (2022). Degradation of curcumin-mediated photodynamic technology (PDT) on polycyclic aromatic hydrocarbons in oysters and toxicity evaluation of PDT-treated oysters. Int. J. Food Sci. Technol..

[9] Long Y.H., Sun Y., Zhou B., Zhu G., Chen X.L., Qi Y.J., Wang K. (2024). Photosensitization of riboflavin reduces the susceptibility to gray mold in postharvest kiwifruit. Postharvest Biol. Technol..

[10] Bhavya M.L., Hebbar H.U. (2019). Sono-photodynamic inactivation of *Escherichia coli* and *Staphylococcus aureus* in orange juice. Ultrason. Sonochem..

[11] Zou Y., Yu Y.S., Cheng L.N., Li L., Zou B., Wu J.J., Zhou W., Li J., Xu Y.J. (2021). Effects of curcumin-based photodynamic treatment on quality attributes of fresh-cut pineapple. LWT-Food Sci. Technol..

[12] Lee I.H., Cho E.R., Kang D.H. (2023). The effect of quercetin mediated photodynamic inactivation on apple juice properties at different temperature and its bactericidal mechanism. Food Control..

[13] Teng X.X., Zhang M., Mujumdar A.S. (2023). Phototreatment (below 1100 nm) improving quality attributes of fresh-cut fruits and vegetables: A review. Food Res. Int..

[14] Lin Y.L., Lai D.N., Wang D.H., Zhou F., Tan B.K., Zhang Z.G., Hu J.M., Lin S.L. (2021). Application of curcumin-mediated antibacterial photodynamic technology for preservation of fresh *Tremella fuciformis*. LWT-Food Sci. Technol..

[15] Dou J.F., Kou X.H., Wu C.E., Fan G.J., Li T.T., Li X.J., Zhou D.D., Yan Z.C., Zhu J.P. (2023). Recent advances and development of postharvest management research for fresh jujube fruit: A review. Sci. Hortic..

[16] Seididamyeh M., Netzel M.E., Mereddy R., Sultanbawa Y. (2024). Curcumin-mediated photodynamic treatment to extend the postharvest shelf-life of strawberries. J. Food Sci..

[17] Prasad A., Wynands E., Roche S.M., Romo-Bernal C., Allan N., Olson M., Levengood S., Andersen R., Loebel N., Sabino C.P. (2024). Photodynamic inactivation of foodborne Bacteria: Screening of 32 potential photosensitizers. Foods.

[18] Song L.L., Zhang F., Yu J.S., Wei C.L., Han Q.M., Meng X.H. (2020). Antifungal effect and possible mechanism of curcumin mediated photodynamic technology against *Penicillium expansum*. Postharvest Biol. Technol..

[19] Yuan Y., Liu Q.Y., Huang Y.J., Qi M.Y., Yan H.Y., Li W.L., Zhuang H. (2022). Antibacterial efficacy and mechanisms of curcumin-based photodynamic treatment against *Staphylococcus aureus* and its application in juices. Molecules..

[20] Huang J.M., Chen B., Li H.H., Zeng Q.H., Wang J.J., Liu H.Q., Pan Y.J., Zhao Y. (2020). Enhanced antibacterial and antibiofilm functions of the curcumin-mediated photodynamic inactivation against *Listeria monocytogenes*. Food Control..

[21] Ni Z.J., Xue Y., Wang W., Du J., Thakur K., Ma W.P., Wei Z.J. (2024). Carbon dots-mediated photodynamic treatment reduces postharvest senescence and decay of grapes by regulating the antioxidant system. Foods.

[22] Elam E., Lv Y.M., Wang W., Thakur K., Ma W.P., Ni Z.J., Wei Z.J. (2022). Effects of nitric oxide on postharvest storage quality of *Lycium barbarum* fruit. Food Sci. Technol..

[23] Liu C.H., Zheng H.H., Sheng K.L., Liu W., Zheng L. (2018). Effects of melatonin treatment on the postharvest quality of strawberry fruit. Postharvest Biol. Technol..

[24] Xing Y.G., Yang H., Guo X.L., Bi X.F., Liu X.C., Xu Q.L., Wang Q., Li W.X., Li X.L., Shui Y.R. (2020). Effect of chitosan/Nano-TiO_2_ composite coatings on the postharvest quality and physicochemical characteristics of mango fruits. Sci. Hortic..

[25] Yang Q.Z., Hou J., Wang F., Qi Y.J., Zhao Q.F. (2025). Cold shock treatment alleviates pitting in sweet cherry fruit by enhancing antioxidant enzymes activity and regulating membrane lipid metabolism. J. Sci. Food Agric..

[26] Liu S.W., Jing G.Q., Zhu S.H. (2022). Nitric oxide (NO) involved in antioxidant enzyme gene regulation to delay mitochondrial damage in peach fruit. Postharvest Biol. Technol..

[27] Lu X.H., Zhang H.J., Zhang N., Dong C.H., Ji H.P., Yu J.Z., Ban Z.J., Yan R.X., Zhang T., Chen C.K. (2023). Effects of ozone treatment on gene profiling involved in ASA-GSH cycle in postharvest cantaloupe. Sci. Hortic..

[28] Zhang Y.T., Li S.L., Deng M.Y., Gui R., Liu Y.Q., Chen X.P., Lin Y.X., Li M.Y., Wang Y., He W. (2022). Blue light combined with salicylic acid treatment maintained the postharvest quality of strawberry fruit during refrigerated storage. Food Chem. X.

[29] Du Y.H., Mi S.N., Wang H.H., Yuan S.F., Yang F.W., Yu H., Xie Y.F., Guo Y.H., Cheng Y.L., Yao W.R. (2024). Intervention mechanisms of cold plasma pretreatment on the quality, antioxidants and reactive oxygen metabolism of fresh wolfberries during storage. Food Chem..

[30] Wang J.J., Zhao L.N.K., Tian W., Zhang H.Y., Wang P., Zhan Q., Fan H.W., Yu X. (2024). Carvacrol maintains antioxidant capacity in goji fruit by increasing the content of bioactive compounds. LWT-Food Sci. Technol..

[31] Patiño L.S., Castellanos D.A., Herrera A.O. (2018). Influence of 1-MCP and modified atmosphere packaging in the quality and preservation of fresh basil. Postharvest Biol. Technol..

[32] Ban Z.J., Wei W.W., Yang X.Z., Feng J.H., Guan J.F., Li L. (2015). Combination of heat treatment and chitosan coating to improve postharvest quality of wolfberry (*Lycium barbarum*). Int. J. Food Sci. Technol..

[33] Chang X.J., Liang Y.G., Guo T.J., Wang Y., Yang J.L. (2023). Combined treatment of acidic electrolyzed water and high-voltage electrostatic field improves the storage quality of huping jujube (*Ziziphus jujuba* Mill. cv. Huping). Foods.

[34] He X.L., Wu C., Lu L., Yan X.X., Yu H., Kang N.B. (2022). Influence of acidic electrolyzed water combined with vacuum precooling treatment on quality and antioxidant performance of fresh *Lycium barbarum* L.. J. Food Process Preserv..

[35] Damas-Job M.D., Soriano-Melgar L.D.A., Rodríguez-Herrera R., Peralta-Rodríguez R.D., Rivera-Cabrera F., Martínez-Vazquez D.G. (2023). Effect of broccoli fresh residues-based extracts on the postharvest quality of cherry tomato (*Solanum lycopersicum* L.) fruits. Sci. Hortic..

[36] Yang J.N., Yin J.T., Wang K., Zhao L., Yang Z.B., Cai Y.T., Lou J.F., Huang C., Shen Q. (2024). Advanced technology in fruit preservation: Effects of nanoscale charged water particles on storage quality and reactive oxygen species in blueberries. Food Res. Int..

[37] Du J., Ni Z.J., Wang W., Thakur K., Ma R.H., Ma W.P., Wei Z.J. (2024). Carbon dot-mediated photodynamic treatment improves the quality attributes of post-harvest Goji Berries (*Lycium barbarum* L.) *via* regulating the antioxidant system. Foods.

[38] Chen Y.H., Hung Y.C., Chen M.Y., Lin M.S., Lin H.T. (2019). Enhanced storability of blueberries by acidic electrolyzed oxidizing water application may be mediated by regulating ROS metabolism. Food Chem..

[39] Hasanuzzaman M., Bhuyan M.H.M.B., Anee T.I., Parvin K., Nahar K., AL Mahmud J., Fujita M. (2019). Regulation of ascorbate-glutathione pathway in mitigating oxidative damage in plants under abiotic stress. Antioxidants.

[40] Zhang X., Wu H., Zhang L.A., Sun Q.J. (2018). Horseradish peroxidase-mediated synthesis of an antioxidant gallic acid-*g*-chitosan derivative and its preservation application in cherry tomatoes. RSC Adv..

[41] Shen A., Zhang T.Z., Li S.Z., Xiao M.R., Tian Z.J., Zhang J., Lu T.T., Yang W.W. (2024). Innovative chitosan-onion polysaccharide composite films: A study on the preservation effects on cherry tomatoes. J. Food Sci..

[42] Li T.T., Yan Z.C., Li Y.F., Kou X.H., Wu C., Xu D.Y., Zhou D.D., Cong K.P., Fan G.J., Li X.J. (2025). Mechanistic insights into the enhancement of storage quality characteristics of fresh goji berry through non-thermal optical treatments (UV-C and IPL). Food Chem..

